# The impact of different body surface area prediction equations on ventricular dilatation prevalence in youth soccer players

**DOI:** 10.3389/fcvm.2025.1627460

**Published:** 2025-09-01

**Authors:** Marco Alessandro Minetto, Elisabetta Toso, Federico Della Vecchia, Andrea Ferraris, Massimo Magistrali, Gianluca Alunni, Chiara Busso, Angelo Pietrobelli, John A. Shepherd, Steven B. Heymsfield

**Affiliations:** ^1^ Division of Physical Medicine and Rehabilitation, Department of Surgical Sciences, University of Turin, Torino, Italy; ^2^ Division of Cardiology, Cardiovascular and Thoracic Department, Città della Salute e della Scienza Hospital, Torino, Italy; ^3^ Juventus Football Club, Torino, Italy; ^4^ Biolab, PolitoBIOMed Lab, Department of Electronics and Telecommunications, Politecnico di Torino, Torino, Italy; ^5^ J|medical, Torino, Italy; ^6^ Paediatric Unit, Department of Surgical Sciences, Dentistry, Gynaecology and Paediatrics, University of Verona, Verona, Italy; ^7^ Pennington Biomedical Research Centre, Baton Rouge, LA, United States; ^8^ Department of Epidemiology, University of Hawaii Cancer Center, Honolulu, HI, United States

**Keywords:** body surface area, non-ischaemic cardiomyopathies, digital anthropometry, soccer, echocardiography

## Abstract

**Background:**

The normalization of echocardiographic variables for body surface area (BSA) enables to obtain relative indexes of ventricular size that are useful for diagnosis and monitoring of non-ischaemic cardiomyopathies. The BSA values commonly considered in the clinical practice are obtained using predictive equations. Our aims were to investigate the accuracy of different predictive equations for BSA estimation and to evaluate the impact of different BSA normalizations on ventricular dilatation prevalence in youth soccer players.

**Methods:**

Two samples of 369 and 111 youth soccer players of both genders were recruited. Acquisition of optical images (for the players of the first sample), two-dimensional echocardiographic assessment (for the players of the second sample), and weight and height measurements (for the players of both samples) were performed. BSA estimates were derived from optical images and from ten different predictive equations obtained from the literature.

**Results:**

In the first sample of 369 players, we found differences among the BSA estimates obtained with ten predictive equations in both male and female players and we also found that all predictive equations in male players and almost all predictive equations in female players overestimated BSA compared to the optical imaging-derived BSA. In the second sample of 111 soccer players, we found that the normalization of each echocardiographic variable for different BSA values resulted in significantly different relative values and that ventricular dilatation prevalence was a function of BSA normalization.

**Conclusion:**

Newly developed equations seemed the most accurate for BSA estimation in both male and female players: therefore, we suggest to adopt these equations for BSA estimation in youth soccer players. The BSA normalization impacts on the ventricular dilatation prevalence: therefore, we suggest to adopt the proper normalization approach to improve the clinical validity of echocardiography in athletes.

## 1 Introduction

Body surface area (BSA) is often used in clinical assessments over body weight because it is a more accurate indicator of metabolic mass (i.e., fat free mass) (1). Moreover, BSA is also associated with the ventricular performance and mass (2): therefore, correction of the cardiac output for BSA can be used to quantify cardiac index, while corrections of ventricular mass (obtained by cardiac magnetic resonance) or size (obtained by echocardiography) for BSA are recommended to obtain relative indexes of ventricular mass and size that improve the clinical validity of imaging techniques. Consistently, previous reports showed that ventricular size can be misclassified if the normalization to BSA is not performed (3). The misclassification may result in under-detection of early-stage non-ischaemic cardiomyopathies, which are rare but recognized causes of sudden cardiac death in athletes (4). Therefore, both absolute and BSA-corrected quantitative measurements are currently available on all echocardiogram reports. The BSA values adopted in these reports are obtained using predictive equations, given the complexity of the direct assessment of BSA through traditional methods of coating, surface integration, and triangulation (the latter method consists in marking the body surface with regular triangular figures and calculating the area of these figures from their linear dimensions). The first equation proposed for BSA estimation was derived by Meeh (5) and only included mass as a predictor. Not long after, DuBois and DuBois proposed a new predictive equation introducing height as a variable (6). Although this equation has been shown to underestimate BSA for low body mass index and to overestimate BSA for high body mass index (2), it continues to be the most commonly used equation in the clinical setting (1, 7, 8). In recent years, many other predictive equations have been proposed (8), but several studies have documented discrepancies among different predictive equations (2, 8–12), thereby highlighting the need to produce (and use in the clinical practice) population-specific equations for BSA prediction. It is worth highlighting that some of the new predictive equations (9, 11, 12) were developed by using a high-resolution, fast, and automatic version of the triangulation procedure such as the 3-dimensional optical imaging (3DOI). During the last decade, optical body scanners became available and can be now used in the clinical setting to obtain a “virtual twin” (i.e., an avatar mesh of the human body) with automated anthropometry (which includes lengths, circumferences, volumes, surface areas, and BSA) (7, 13–15). To our knowledge, none of the previous studies investigating BSA predictive equations (by using either a traditional direct assessment of BSA or a digital anthropometric approach) was performed in athletes, with the exception of the study by Daniell et al. (10) who compared in 1,452 healthy subjects (from the general Australian population) and 262 athletes (competitive rowers) a criterion BSA measurement obtained from a 3D whole body laser scanning and BSA estimations obtained from fifteen different predictive equations. They found that the equation by Shuter (16) was the most accurate to be used for BSA prediction in a Western population of young (aged 18–30 years) subjects. We hypothesized that the comparison between 3DOI-derived BSA and estimates obtained with different predictive equations can enable to identify the most accurate equation(s) to be used for BSA prediction in youth soccer players. We also hypothesized that equations producing an overestimation of BSA could impact on the relative values of echocardiographic variables commonly used to assess the ventricular size, thereby resulting in a misclassification of the ventricular dilation. Therefore, the aims of this study were to investigate the accuracy of different predictive equations for BSA estimation in a large group of youth soccer players and to evaluate the impact of different BSA normalizations on ventricular dilatation prevalence in another group of soccer players.

## 2 Methods

### 2.1 Participants and protocol

The study setting was a sports medicine and rehabilitation center where a convenience sample of 254 male soccer players [median age (1st–3rd quartile): 16.2 (15.0–18.1) years; body mass index: 21.4 (20.1–22.5) kg/m^2^] and 115 female soccer players [age: 16.0 (14.9–17.0) years; body mass index: 20.8 (19.7–21.8) kg/m^2^] were recruited to participate.

Another convenience sample of 86 male soccer players [median age (1st–3rd quartile): 19.5 (18.0–24.0) years; body mass index: 23.1 (22.4–24.0) kg/m^2^] and 25 female soccer players [age: 23.0 (19.0–26.0) years; body mass index: 22.7 (20.9–23.1) kg/m^2^] was also recruited.

Both samples consisted of players with training history and volume greater than 5 years and 10 h per week (including technical sessions, aerobic training reaching at least 75% of the maximal heart rate, and strength training), respectively.

The single study visits were performed as part the preseason investigations and included acquisition of optical images (for the players of the first sample), two-dimensional echocardiographic assessment (for the players of the second sample), and weight and height measurements (for the players of both samples).

All subjects (or their parents in case of underage subjects) gave their written consent after receiving a detailed explanation of the protocol. The study conformed to the guidelines of the Declaration of Helsinki and was approved by the ethics committee of the University of Turin (protocol n. 0115311).

### 2.2 Measurements

Body weight and height were measured while each player was dressed in undergarments and with bare feet. Body weight and height were measured (to the nearest 0.1 kg and 0.5 cm, respectively) using a standard scale with stadiometer (model Seca 799, Seca GmbH & Co. Kg, Hamburg, Germany).

**Figure 1:**
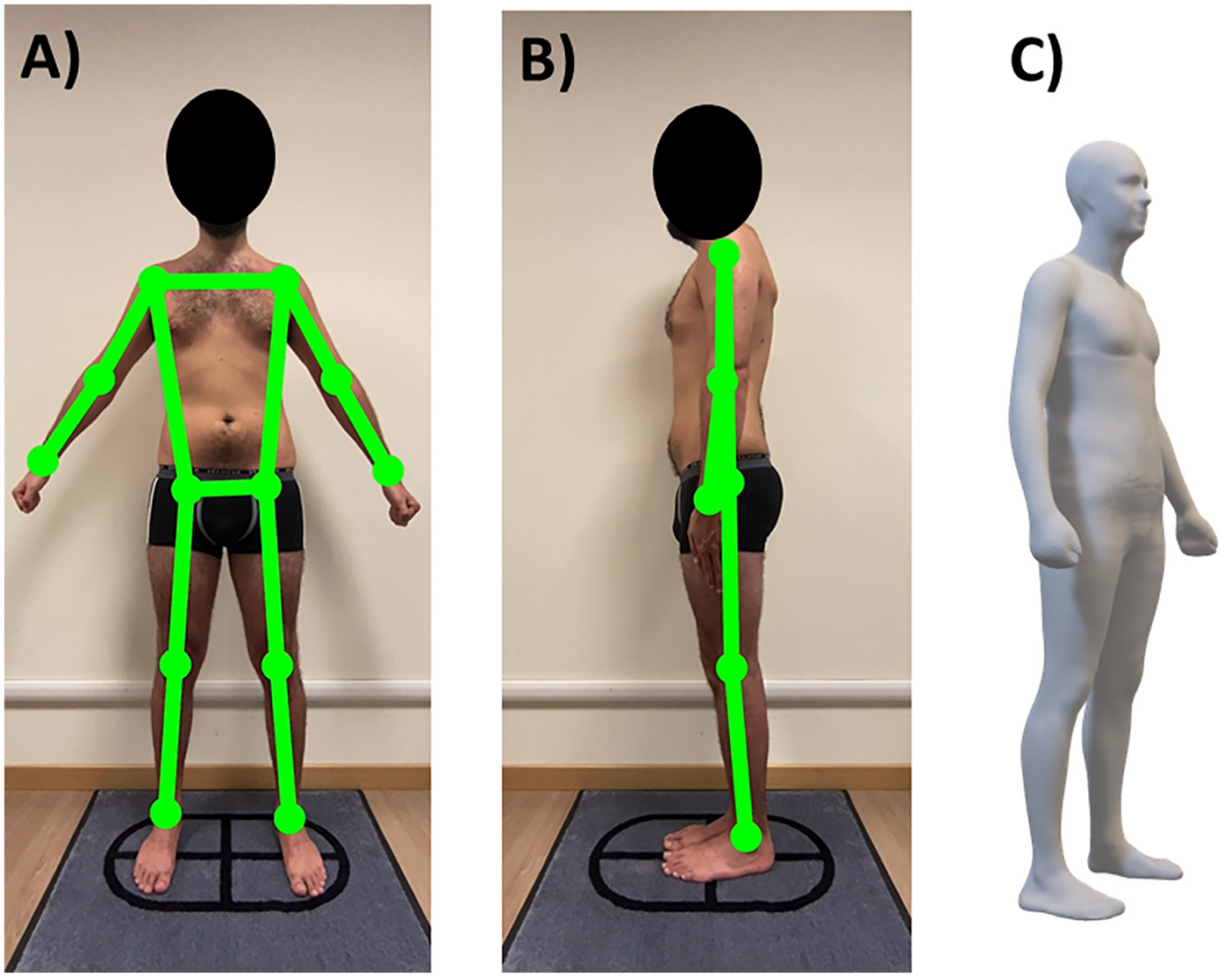
Representative example of acquisition of the frontal **(A)** and lateral **(B)** images in a male soccer player (the investigated subject gave his written consent for study participation and publication of anonymized images) and the relative avatar **(C)**.

Optical images were taken with Mobile Fit app (version 3.0, Size Stream LLC, Cary, NC, USA) installed on an iPad (Apple Inc, Cupertino, CA, USA): this 3D scanning mobile application was specifically selected because of previous studies showing its accuracy and reproducibility for body size and composition assessment (17–24). Optical images were acquired using a standardized protocol, as previously described (25). Voice commands from the app guided each player into position for the self-scan: the player was asked to assume a “front A-pose” (and to maintain the pose without movements of the trunk or limbs) to capture the frontal image (Figure 1A). Next, the player was asked to assume a “side pose” to capture the lateral image (Figure 1B). After the image capture, the app software generated a de-identified 3D humanoid avatar (Figure 1C: point clouds are converted to a mesh connected by triangles with approximately 50,000 vertices and 100,000 faces) and automatically quantified the BSA (as the sum of surface area of all triangles of the mesh). The acquisition of the frontal and lateral images was performed in duplicate to obtain two avatars for each player: the average BSA of the two avatars was considered for further analyses.

Cardiac structure was assessed with transthoracic echocardiography and Doppler imaging (Vivid E9 with Xdclear, GE Healthcare, Boston, MA, USA), according to a standardized clinical protocol (26). Two-dimensional echocardiography was employed: parasternal long axis view and apical view measures were performed according to the American Society of Echocardiography Guidelines (27). All ultrasound procedures were performed and analysed by two independent cardiologists (ET, GA). Parameters assessed were left ventricular end-diastolic diameter (LVEDD: absolute and relative, normalized to BSA), left ventricular end diastolic volume (LVEDV: absolute and relative, normalized to BSA), and right ventricular basal diameter (RVBD: absolute and relative, normalized to BSA). The left ventricular dilatation was identified if one or both the relative variables were above the following cut-points: (i) LVEDD: 30 mm/m^2^ for males and 31 mm/m^2^ for females (27); (ii) LVEDV: 74 ml/m^2^ for males and 61 ml/m^2^ for females (27). The right ventricular dilatation was identified if RVBD was above the cut-point of 22 mm/m^2^ for both males and females (28). The occurrence of both left and right ventricular dilation was not included in the calculation of the ventricular dilation prevalence in order to exclude possible cases of “athlete's heart” (i.e., the training-induced physiological left and right ventricular hypertrophy resulting in a balanced enlarged heart) (29).

### 2.3 BSA predictive equations

We applied ten different equations obtained from the literature to predict BSA from weight alone (30) and from weight and height (6, 9, 11, 12, 16, 31–34). These equations were selected due to their use in clinical applications and citation in clinical manuscripts (Table 1).

**Table 1:** Body surface area (BSA) predictive equations for comparison with 3-dimensional optical imaging-derived BSA estimates.

Authors	Equation	References
Eq. **#** 1 (DuBois)	BSA = 0.007184 × weight ^0.425^ × height ^0.725^	(6)
Eq. **#** 2 (Sendroy)	BSA = 0.0097×(weight + height) - 0.545	(31)
Eq. **#** 3 (Gehan)	BSA = 0.0235 × weight ^0.51456^ × height ^0.42246^	(32)
Eq. **#** 4 (Mosteller)	BSA = 0.016667 × weight ^0.5^ × height ^0.5^	(33)
Eq. **#** 5 (Shuter)	BSA = 0.00949 × weight ^0.441^ × height ^0.655^	(16)
Eq. **#** 6 (Tikuisis)	Male BSA = 0.01281 × weight ^0.44^ × height ^0.60^ Female BSA = 0.01474 × weight ^0.47^ × height ^0.55^	(9)
Eq. **#** 7 (Livingston)	BSA = 0.1173 × weight ^0.6466^	(30)
Eq. **#** 8 (Schlich)	Male BSA = 0.000579479 × weight ^0.38^ × height ^1.24^ Female BSA = 0.000975482 × weight ^0.46^ × height ^1.08^	(34)
Eq. **#** 9 (Kuehnapfel)	BSA = 0.015 × weight ^0.4259^ × height ^0.5751^	(11)
Eq. **#** 10 (Ashby-Thompson)	Male BSA = 0.01624 × weight ^0.4725^ × height ^0.5231^ Female BSA = 0.01522 × weight ^0.4921^ × height ^0.5231^	(12)

Units of measurements: kg for weight; cm for height.

### 2.4 Statistical analysis

Prior to statistical analyses, outliers of 3DOI-derived BSA estimation were assessed using the Grubbs’ outlier test (alpha = 0.05) (35) as a part of preprocessing quality control: no players were identified as outliers (i.e., no measurements were removed).

Normality of the data distributions was assessed with the Shapiro–Wilk test and parametric statistical tests (paired sample T test, one-way repeated measures ANOVA, Pearson correlation analysis) or non-parametric tests (Mann–Whitney *U* test, Friedman's ANOVA) were adopted. The following comparisons between 3DOI – derived BSA and BSA estimates obtained with the ten predictive equations were also performed: (i) root mean square error (RMSE); (ii) relative bias (quantified as equation-derived estimates minus the 3DOI-derived estimates and expressed as %) and relative standard deviation (SD) of the differences; (iii) absolute average differences obtained from the Bland-Altman plots. Moreover, the McNemar's test was adopted to compare paired proportions.

Data were expressed as median and 1st–3rd quartile and were represented with violin plots. The threshold for statistical significance was set to *P* = 0.05. Statistical tests were performed with Prism v. 9.0 (GraphPad Software LLC, Boston, MA, USA), MedCalc v. 20.218 (MedCalc Software Ltd, Ostend, Belgium), and SPSS v. 20.0 (SPSS Inc., Chicago, IL, USA) software packages.

## 3 Results

### 3.1 Accuracy of BSA predictive equations

**Figure 2:**
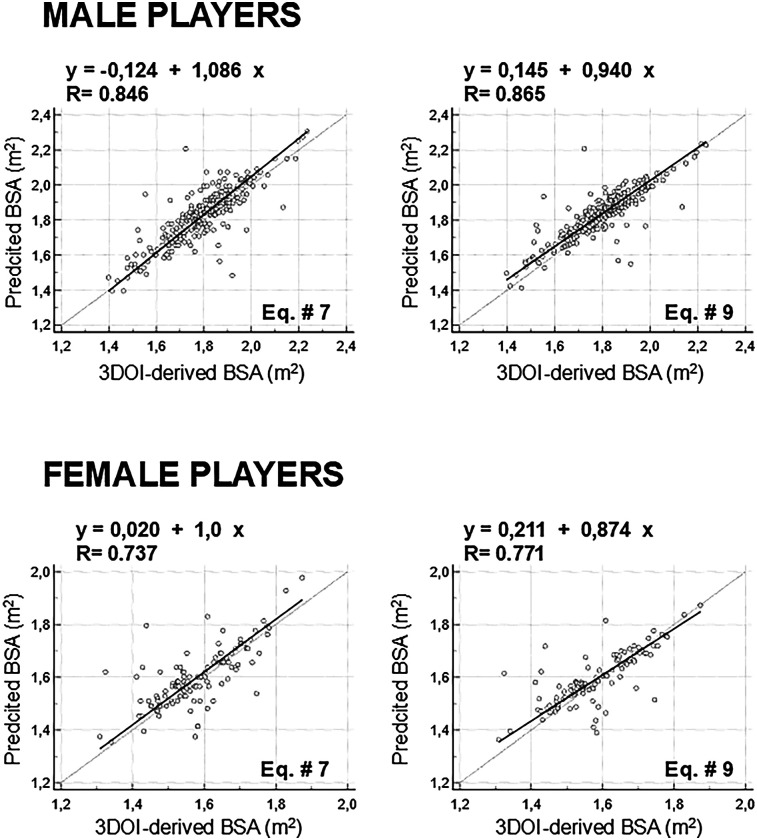
Top panels: correlations between 3-dimentional optical imaging (3DOI)-derived body surface area (BSA) and predicted BSA estimates showing the lowest (BSA predicted through Eq. # 7 by Livingston: left panels) and highest (BSA predicted through Eq. # 9 by Kuehnapfel: right panels) value of R in males (top panels) and females (bottom panels).

**Figure 3:**
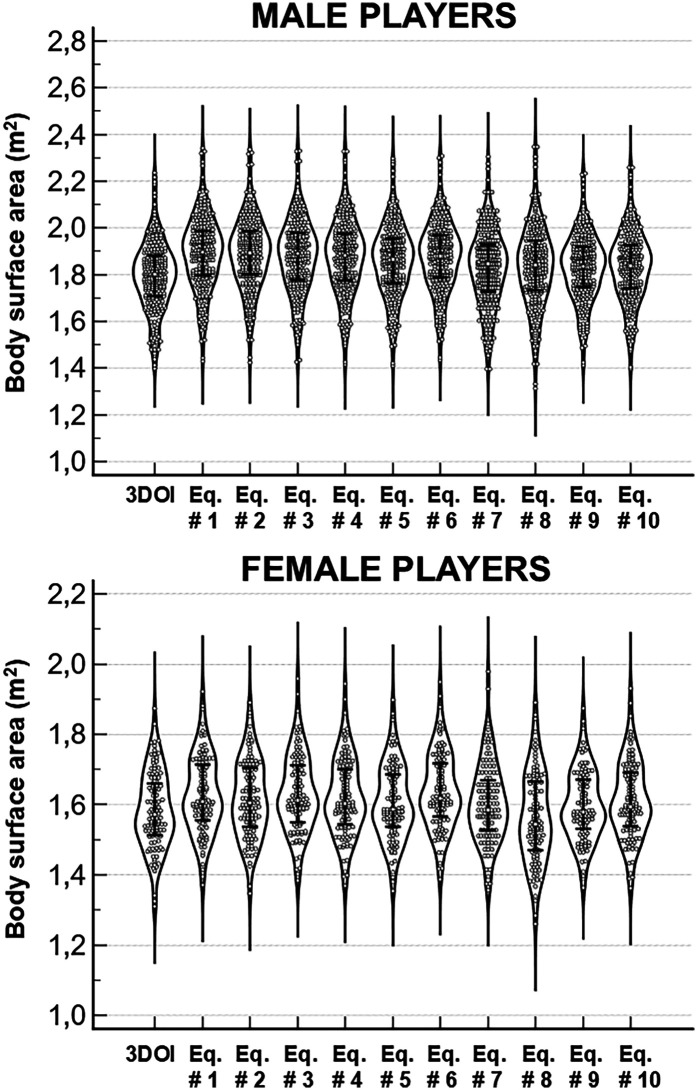
Violin plots of the body surface area (BSA) estimates obtained with the 3-dimentional optical imaging (3DOI) and with ten predictive equations in the group of 396 players (254 males and 115 females). Error bars indicate the median values and the interquartile ranges.

Table 2 and Figures 2, 3 show the comparisons between 3DOI – derived BSA and BSA estimates obtained with the ten predictive equations in the whole group of 369 soccer players (254 males and 115 females). Although 3DOI – derived BSA and predicted BSA estimates were highly correlated in males (as shown in Table 2, Pearson correlation coefficients were in the range 0.846–0.865: top panels of Figure 2 show the correlations with the lowest and highest value of R), significant differences (one-way repeated measures ANOVA: F = 319.0; *P* < 0.0001) were obtained in males between 3DOI-derived BSA and all predicted BSA estimates (Table 2; Figure 3: *P* < 0.0001 for all comparisons). Although 3DOI—derived BSA and predicted BSA estimates were highly correlated also in females (as shown in Table 2, Pearson correlation coefficients were in the range 0.737–0.771: bottom panels of Figure 2 show the correlations with the lowest and highest value of R), significant differences (F = 78.0; *P* < 0.0001) were obtained in females between 3DOI-derived BSA and most of the predicted BSA estimates (Table 2; Figure 3: *P* < 0.0001 for six comparisons and *P* < 0.05 for one comparison), with the exclusion of the BSA estimates obtained with the Eq. # 7 by Livingston, Eq. # 8 by Schlich, and Equation 9 by Kuehnapfel. Briefly, all predictive equations in male players and almost all predictive equations in female players overestimated BSA compared to the criterion method.

**Table 2:** Median (1st–3rd quartile) values of body surface area (BSA) estimates obtained in the sample of 369 soccer players (254 males and 115 females) with three-dimensional optical imaging (3DOI-derived BSA: first row) and with ten predictive equations.

BSA values	MALES (*n* = 254)				FEMALES (*n* = 115)			
	Median (1st–3rd quartile) (m^2^)	PCC	RMSE (m^2^)	Bias ± SD of differences (%)	Median (1st–3rd quartile) (m^2^)	PCC	RMSE (m^2^)	Bias ± SD of differences (%)
3DOI-derived BSA	1.802 (1.707–1.882)	–	–	–	1.572 (1.515–1.658)	–	–	–
Eq. # 1 (DuBois)	1.897** (1.797–1.988)	0.865	0.125	5.5 ± 4.5	1.608** (1.555–1.713)	0.771	0.086	3.0 ± 4.9
Eq. # 2 (Sendroy)	1.892** (1.802–1.985)	0.863	0.122	5.4 ± 4.5	1.604** (1.536–1.703)	0.759	0.082	2.2 ± 5.0
Eq. # 3 (Gehan)	1.891** (1.778–1.979)	0.862	0.116	4.8 ± 4.6	1.612** (1.552–1.710)	0.767	0.088	3.1 ± 4.9
Eq. # 4 (Mosteller)	1.883** (1.775–1.974)	0.865	0.113	4.5 ± 4.6	1.602** (1.547–1.698)	0.770	0.082	2.5 ± 4.9
Eq. # 5 (Shuter)	1.846** (1.727–1.927)	0.865	0.101	3.7 ± 4.5	1.586* (1.536–1.685)	0.770	0.075	1.5 ± 4.8
Eq. # 6 (Tikuisis)	1.880** (1.787–1.968)	0.865	0.113	4.7 ± 4.5	1.618** (1.569–1.717)	0.770	0.092	3.7 ± 4.9
Eq. # 7 (Livingston)	1.846** (1.727–1.927)	0.846	0.093	1.9 ± 4.9	1.584 (1.528–1.665)	0.737	0.082	1.4 ± 5.3
Eq. # 8 (Schlich)	1.839** (1.734–1.943)	0.861	0.096	2.2 ± 5.0	1.543 (1.417–1.666)	0.767	0.084	-1.2 ± 5.3
Eq. # 9 (Kuehnapfel)	1.834** (1.747–1.916)	0.865	0.083	2.2 ± 4.3	1.579 (1.533–1.670)	0.771	0.071	1.0 ± 4.7
Eq. # 10 (Ashby-Thompson)	1.838** (1.740–1.924)	0.864	0.086	2.2 ± 4.4	1.593** (1.541–1.690)	0.770	0.079	2.0 ± 4.9

PCC, pearson correlation coefficient; RMSE, root mean square error; SD, standard deviation.

** Significantly different from 3DOI at *P* < 0.0001.

* Significantly different from 3DOI at *P* < 0.05.

As shown in Table 2, Eq. # 9 by Kuehnapfel ranked first for the lowest RMSE in both male and female players (respectively, 0.083 m^2^ and 0.071 m^2^), while the less accurate equations (i.e., highest RMSE) were Eq. # 1 by DuBois in male players (0.125 m^2^) and Eq. # 6 by Tikuisis in female players (0.092 m^2^).

**Figure 4:**
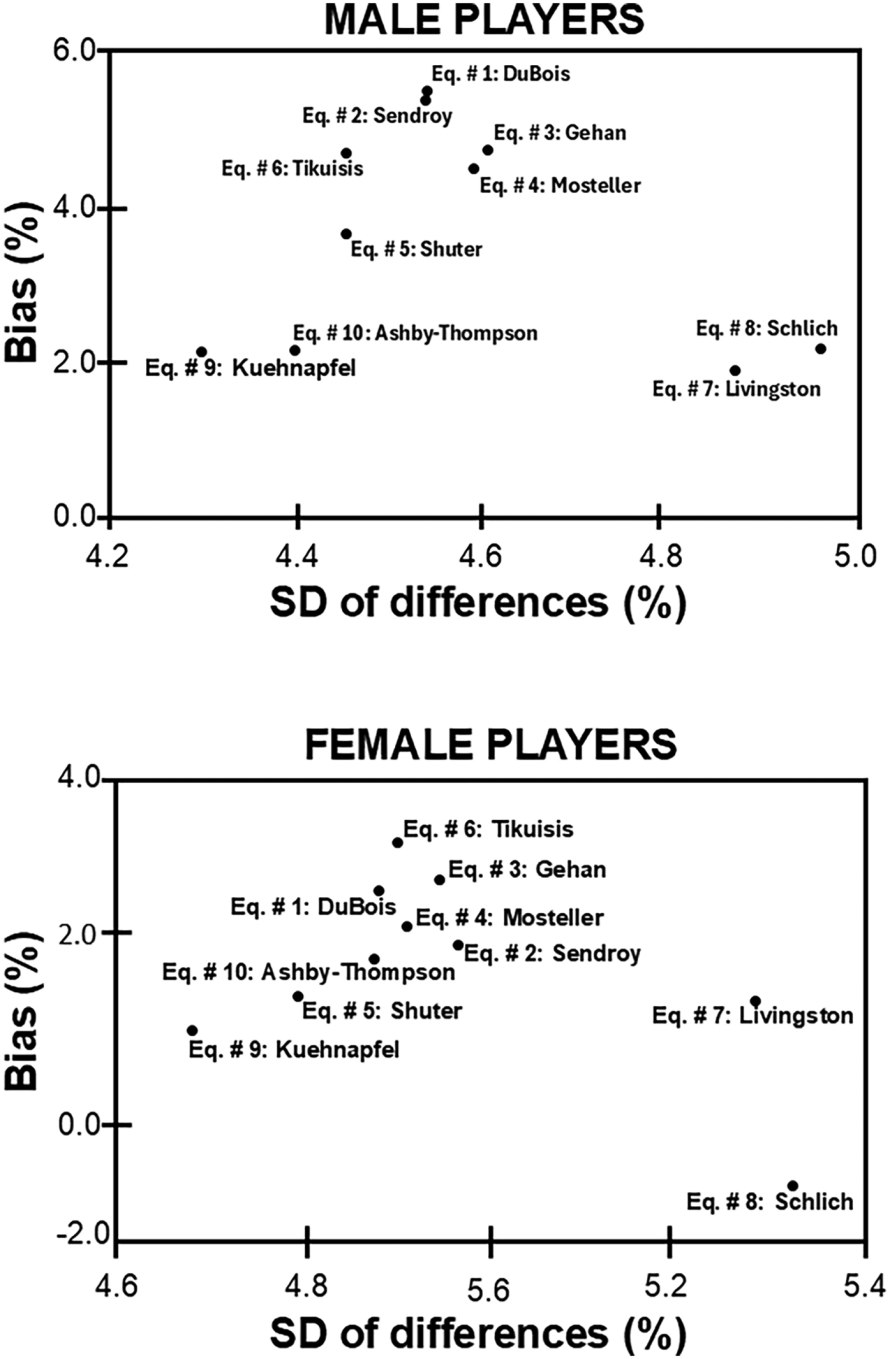
Relative bias and relative standard deviation (SD) of the differences between the 3-dimentional optical imaging-derived surface areas and those obtained by ten predictive equations.

Figure 4 shows for all equations the average relative bias plotted against the standard deviation of the differences (values are reported in Table 2). In male players, Eq. # 5 by Shuter, Eq. # 9 by Kuehnapfel, and Eq. # 10 by Ashby-Thompson showed high accuracy (i.e., low systematic error) and low SD of differences (i.e., low random error), while Eq. # 1 by DuBois (average bias: 5.5%) was the less accurate equation for the dataset of male subjects analysed in this study. In female players, the same three equations (Eq. # 5 by Shuter, Eq. # 9 by Kuehnapfel, and Eq. # 10 by Ashby-Thompson) showed high accuracy (i.e., low systematic error) and low SD of differences (i.e., low random error), while Eq. # 6 by Tikuisis (average bias: 3.7%) was the less accurate equation for the dataset of female subjects analysed in this study.

The Bland-Altman analyses of the three equations (Eq. # 5 by Shuter, Eq. # 9 by Kuehnapfel, and Eq. # 10 by Ashby-Thompson) presenting high accuracy and low random error showed that the absolute values of the mean differences between 3DOI-derived BSA and predicted BSA estimates ranged between −0.06 m^2^ and −0.04 m^2^ in male players and between −0.01 m^2^ and −0.03 m^2^ in female players (Supplementary Figure S1). Moreover, no significant (*P* > 0.05 for all analyses) positive correlations (i.e., no proportional biases) were observed between the differences and means of the 3DOI-derived BSA and predicted BSA estimates.

### 3.2 BSA estimates and echocardiographic assessments

**Figure 5:**
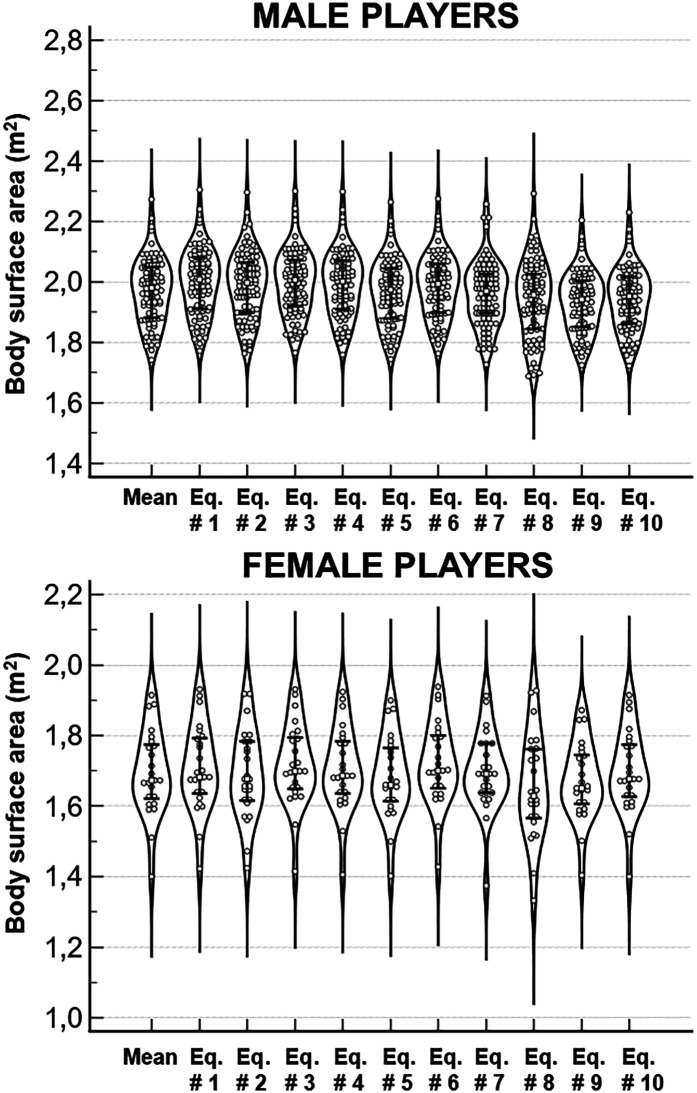
Violin plots of the body surface area (BSA) estimates obtained with ten predictive equations in the group of 111 players (86 males and 25 females). The first column in each plot shows the means of ten predictive equations. Error bars indicate the median values and the interquartile ranges.

Figure 5 shows the predicted BSA values of the whole group of 111 soccer players (86 males and 25 females): significant differences were obtained in both males (Friedman's ANOVA: F = 629.9; *P* < 0.0001) and females (Friedman's ANOVA: F = 115.7; *P* < 0.0001) among the BSA estimates obtained with the ten predictive equations. *Post-hoc* analysis in males showed that the BSA estimates obtained with the Eq. # 9 by Kuehnapfel were significantly lower than the BSA estimates obtained with all other equations (*P* < 0.0001 vs. Eq. # 1 by DuBois, Eq. # 2 by Sendroy, Eq. # 3 by Gehan; Eq. # 4 by Mosteller, Eq. # 5 by Shuter, Eq. # 6 by Tikuisis, Eq. # 7 by Livingston; *P* = 0.02 vs. Eq. # 8 by Schlich), with the exclusion of the estimates obtained with Eq. # 10 by Ashby-Thompson (*P* = 0.33). *Post-hoc* analysis in females showed that the BSA estimates obtained with the Eq. # 9 by Kuehnapfel were significantly lower than the BSA estimates obtained with all other equations (*P* = 0.01 vs. # Equation 2 by Sendroy; *P* < 0.01 vs. Eq. # 10 by Ashby-Thompson; *P* < 0.0001 vs. Eq. # 1 by DuBois, Eq. # 3 by Gehan; Eq. # 4 by Mosteller, Eq. # 6 by Tikuisis), with the exclusion of the estimates obtained with the Eq. # 5 by Shuter (*P* = 0.75) and Eq. # 8 by Schlich (*P* = 0.99).

Table 3 shows the absolute and relative values of LVEDD, LVEDV, and RVBD obtained in the whole group of 111 players. The absolute values of the three variables were significantly (Mann–Whitney U test: *P* < 0.01 for all comparisons) higher in male players compared with female players. Predicted BSA values (the means of the ten predictive equations were considered for males and females: the violin plots of these two mean distributions are shown in Figure 5) were also significantly (Mann–Whitney U test: *P* < 0.0001) higher in male players compared with female players [1.980 (1.883–2.049) m^2^ vs. 1.672 (1.627–1.771) m^2^]. Relative values of LVEDD were significantly (Mann–Whitney *U* test: *P* < 0.0001) higher in females compared with males for all BSA normalizations, while no significant differences between males and females were observed for the relative values of LVEDV and RVBD. Friedman's ANOVA showed significant differences both in male players and in female players among the ten relative values of LVEDD (respectively, F = 512.2, *P* < 0.0001 in males and F = 107.2, *P* < 0.0001 in females), LVEDV (respectively, F = 384.0, *P* < 0.0001 and F = 113.1, *P* < 0.0001), and RVBD (respectively, F = 548.8, *P* < 0.0001 and F = 108.3, *P* < 0.0001). Briefly, the correction of each echocardiographic variable for different BSA values resulted in significantly different relative values. Consistently, the ventricular dilatation prevalence was a function of BSA normalization. For example, the normalization of the LVEDV of 147 ml obtained in a male athlete (weight of 77 kg and height of 185 cm) resulted in a relative index of 76.0 ml/m^2^ (by using the BSA estimated with Eq. # 9 by Kuehnapfel: 1.933 m^2^) and of 73.3 ml/m^2^ (by using the BSA estimated with Eq. # 1 by DuBois: 2.004 m^2^) and a different classification concerning the presence of left ventricular dilatation (normalized LVEDV > 74 ml/m^2^). The normalization effect was also evident by using the echocardiographic nomograms for left ventricular dimensions (considering echocardiographic variables, age, and BSA) published for Caucasian youth soccer players (36). In the above-mentioned (16-year-old) athlete presenting with BSA of 1.933 m^2^ (estimated with Eq. # 9 by Kuehnapfel) or 2.004 m^2^ (estimated with Eq. # 1 by DuBois), a measured LVEDD of 65 ml corresponded to Z-score values of 2.05 or 1.95, respectively. Only the former value (>2) can be considered indicative for left ventricular dilatation. Similar to this example, also in the whole group of players the corrections for BSA values predicted through the Eq. # 5 by Shuter, Eq. # 9 by Kuehnapfel, and Eq. # 10 by Ashby-Thompson produced prevalences of ventricular dilatation (respectively, 26.1–27.9–27.0%) significantly (*P* < 0.05 for all comparisons between paired proportions) higher compared with corrections for BSA values predicted through the Eq. # 1 by Dubois, Eq. # 3 by Gehan, Eq. # 4 by Mosteller, Eq. # 6 by Tikuisis (dilatation prevalences: 19.8%–20.7%–20.7%–20.7%, respectively). This normalization effect resulted from differences among the different relative values in the prevalence of both left and right ventricular dilatation. As shown in Table 3, the corrections for BSA values predicted through the Eq. # 5 by Shuter, Eq. # 9 by Kuehnapfel, and Eq. # 10 by Ashby-Thompson identified the left and right ventricular dilatation in 24 and 5–7 players, respectively, while corrections for BSA values predicted through the Eq. # 1 by DuBois identified the left and right ventricular dilatation in 21 and 1 players, respectively.

**Table 3:** Median (1st–3rd quartile) values of absolute and relative (normalized to body surface area—BSA) echocardiographic parameters obtained in the sample of 111 players (86 males and 55 females).

Absolute and relative values	LVEDD		LVEDV		RVBD		Dilatation prevalence: cases/total (%)	Left (right) ventricular dilatation: cases
	Males	Females	Males	Females	Males	Females		
Absolute value	52 (50–54)	49** (47–52)	131 (111.5–150)	105** (97–119)	39 (34–41)	33** (31.3–36.3)		
Relative value—BSA by Eq. # 1 (DuBois)	26.1 (25.4–27.4)	29.8*** (26.9–31.3)	66.5 (55.1–73.4)	65.2 (57.2–70.4)	19.4 (17.3–20.7	19.5 (17.0–21.2)	22/111 (19.8%)	21 (1)
Relative value—BSA by Eq. # 2 (Sendroy)	26.3 (25.5–27.7)	30.2*** (27.3–31.5)	67.2 (56.7–73.7)	66.7 (58.1–70.9)	19.4 (17.3–20.7)	19.6 (17.2–21.5)	25/111 (22.5%)	23 (2)
Relative value—BSA by Eq. # 3 (Gehan)	26.1 (25.5–27.4)	29.5*** (26.9–31.2)	66.5 (57.0–73.9)	64.6 (56.7–70.2)	19.4 (17.2–20.6)	19.5 (16.8–20.8)	23/111 (20.7%)	22 (1)
Relative value—BSA by Eq. # 4 (Mosteller)	26.2 (25.6–27.5)	29.8** (27.0–31.4)	66.8 (57.1–74.0)	64.8 57.2–70.7)	19.4 (17.2–20.7)	19.7 (17.0–21.2)	23/111 (20.7%)	22 (1)
Relative value—BSA by Eq. # 5 (Shuter)	26.6 (25.8–27.8)	30.2** (27.3–31.7)	68.0 (57.5–74.8)	65.9 (58.0–71.4)	19.7 (17.6–21.0)	19.8 (17.2–21.5)	29/111 (26.1%)	24 (5)
Relative value—BSA by Eq. # 6 (Tikuisis)	26.3 (25.5–27.6)	29.5** (26.7–31.1)	67.3 (57.1–74.2)	64.2 (56.6–69.8)	19.5 (17.5–20.8)	19.5 (16.9–21.0)	23/111 (20.7%)	22 (1)
Relative value—BSA by Eq. # 7 (Livingston)	26.8 (25.9–27.9)	29.6*** (27.5–31.3)	67.0 (58.8–75.8)	65.5 (57.1–70.4)	19.9 (17.7–21.0)	20.0 (17.5–21.1)	27/111 (24.3%)	24 (3)
Relative value—BSA by Eq. # 8 (Schlich)	27.0 (26.0–28.5)	30.6** (27.7–32.3)	67.1 (57.0–75.1)	68.3 (59.9–74.1)	19.8 (17.7–21.2)	20.1 (17.5–21.9)	33/111 (29.7%)	25 (8)
Relative value—BSA by Eq. # 9 (Kuehnapfel)	27.0 (26.2–28.4)	30.4** (27.6–31.9)	69.1 (58.6–76.2)	66.3 (58.3–71.5)	20.1 (17.8–21.3)	20.0 (17.4–21.7)	31/111 (27.9%)	24 (7)
Relative value—BSA by Eq. # 10 (Ashby-Thompson)	26.8 (26.0–28.1)	29.7*** (27.0–31.5)	68.6 (58.5–75.9)	65.1 (57.5–71.0)	20.0 (17.9–21.3)	19.8 (17.1–21.3)	30/111 (27.0%)	24 (6)

LVEDD, left ventricular end diastolic diameter (absolute value: mm; relative value: mm/m^2^); LVEDV, left ventricular end diastolic volume (absolute value: ml; relative value: ml/m^2^); RVBD, right ventricular basal diameter (absolute value: mm; relative value: mm/m^2^).

* Significantly different from males at *P* < 0.05.

** Significantly different from males at *P* < 0.01.

*** Significantly different from males at *P* < 0.001.

## 4 Discussion

In the present study we investigated the accuracy of different predictive equations for BSA estimation in a sample of 369 youth soccer players. Moreover, we evaluated the impact of different BSA normalizations on the detection of the left or right ventricular dilatation in another sample of 111 youth soccer players.

We found differences among the BSA estimates obtained with ten predictive equations in both male and female players, in agreement with previous studies (2, 8–12). We also found that all predictive equations in male players and almost all predictive equations in female players overestimated BSA compared to the criterion method. This demonstration of BSA overestimation with predictive equations compared to the criterion method is in agreement with previous findings (2) and highlights the need to use a 3DOI approach for an accurate BSA assessment in athletes. The unavailability of 3D imaging systems and the apparent complexity of their use in the clinical settings can represent barriers to their adoption in the clinical practice. However, recent technological advancements enable the generation of a 3D avatar also from 2D images that can be captured through mobile applications (such as the Mobile Fit app adopted in the present study) already commercially available for tablets and smartphones (17–25).

The alternative and simpler approach for BSA estimation systematically adopted in the clinical practice is based on the use of predictive equations. To our knowledge, this study is the first that compared the accuracy of different predictive equations in a large sample (*n* = 369) of soccer players. We showed that newly developed equations (Eq. # 5 by Shuter, Eq. # 9 by Kuehnapfel, and Eq. # 10 by Ashby-Thompson) had high accuracy (i.e., low systematic error), low SD of differences (i.e., low random error), and no proportional biases in both male and female players, while other new (Eq. # 6 by Tikuisis) or classical (Eq. # 1 by DuBois) equations showed low accuracy in females and males, respectively, compared to the criterion method. Differences in body composition and shape between the groups of adult sedentary subjects previously investigated and our group of youth athletes are possible explanations for the observed BSA overestimation with predictive equations. A methodological implication of these findings is that the users of BSA predictive equations must know the condition for which the selected equations are valid. In other words, the use of population-specific equations is recommended for different clinical applications.

We also found in the other sample of 111 soccer players that the normalization of each echocardiographic variable for different BSA values resulted in significantly different relative values and that the ventricular dilatation prevalence was a function of BSA normalization. In fact, the relative indexes of left and right ventricular size (diameter and/or volume) obtained after normalization for BSA values predicted through newly developed (most accurate) equations produced prevalences of ventricular dilatation significantly higher compared with relative indexes obtained after normalization for BSA values predicted through most of the other (less accurate) equations, in agreement with previous findings (2). Another result of our study that confirms and extends previous findings (37, 38) was that the absolute values of the investigated echocardiographic variables (LVEDD, LVEDV, and RVBD) were significantly higher in male players compared with female players. However, the relative values of LVEDD were significantly higher in females compared with males (for all BSA normalizations). Consistently, Finocchiaro et al. (38) also found absolute values of LVEDD lower in female athletes than in male athletes: however, when the LV measurements were normalized to BSA, women exhibited a higher ventricular size. These authors suggested that a possible mechanism underlying the observed difference between males and females is an inter-gender variability in the exercise-related cardiac remodeling and recommended the use of relative ventricular dimensions to distinguish physiological adaptations from pathology (38).

### 4.1 Limitations

This study has a few limitations that warrant consideration. First, the use of convenient samples introduces the possibility of selection bias, which may influence the generalizability of the findings. Second, the generation of a 3D avatar from 2D images can produce reconstructions less accurate than those obtained by 3D imaging systems (25), thereby affecting the accuracy of the BSA estimation. Third, the potential misclassification of ventricular size due to BSA overestimation was not confirmed by a second-level imaging technique such as cardiac magnetic resonance that could detect false negatives and false positives identified on the basis of relative indexes of ventricular size obtained through different BSA predictive equations. Fourth, we normalized the cardiac size for BSA, but this may be the most accurate method of scaling the ventricular size in athletes (38). In fact, other investigators considered the normalization for height, lean body mass, and allometric models (39, 40). However, we aimed to evaluate the impact of different BSA normalizations on ventricular dilatation prevalence since most American and European guidelines suggest reference values of echocardiographic variables normalized per BSA (38).

## 5 Conclusion

We found differences among the BSA estimates obtained with ten predictive equations in a large sample (*n* = 396) of male and female soccer players. We also showed that all predictive equations in male players and almost all predictive equations in female players overestimated BSA compared to the criterion method. Newly developed equations seemed the most accurate for BSA estimation in both male and female players. Therefore, these equations should be adopted for BSA estimation in youth soccer players. Moreover, we showed that the normalization of echocardiographic variables of ventricular size for different BSA values resulted in significantly different relative values in 111 soccer players and that ventricular dilatation prevalence in this sample was a function of BSA normalization. Therefore, the proper normalization approach should be adopted to improve the clinical validity of echocardiography in athletes.

## Data Availability

The raw data supporting the conclusions of this article will be made available by the authors, without undue reservation.

## Supplementary material

Supplementary Figure S1 Bland-Altman plots of differences vs. means of the body surface area (BSA) estimates obtained with the 3-dimentional optical imaging (3DOI) and with the following three predictive equations: Eq. # 5 by Shuter, Eq. # 9 by Kuehnapfel, and Eq. # 10 by Ashby-Thompson. In each plot, the solid horizontal line depicts the mean of the differences, whereas dashed horizontal lines represent the upper and lower limit of agreement (SD: standard deviation of the differences). The error bar displayed on each horizontal line represents the 95% confidence interval of the corresponding quantity. The dashed-dotted linear regression line (sandwiched between its 95% confidence interval curves) showed no proportional bias (its slope is not different from zero) for all equations.
