# Supplementary figures and images for: The impact of different body surface area prediction equations on ventricular dilatation prevalence in youth soccer players
Source: Front Cardiovasc Med. 2025 Sep 1;12:1627460. doi: 10.3389/fcvm.2025.1627460 (PMC12434081; doi:10.3389/fcvm.2025.1627460)

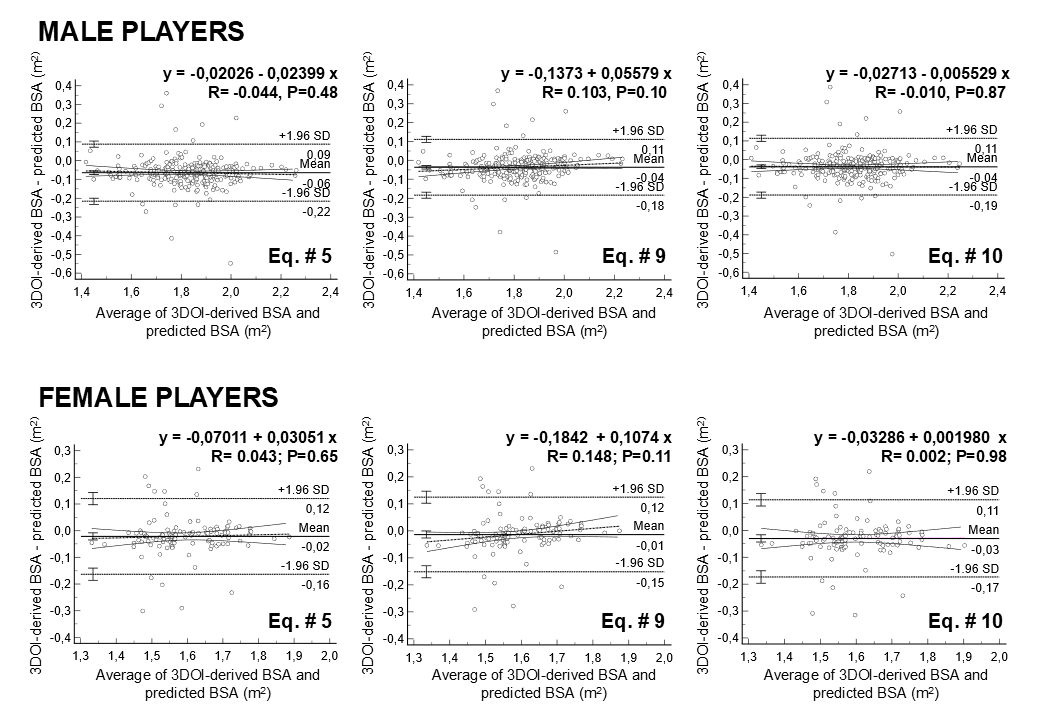

Supplement: Supplementary Figure S1 — Bland-Altman plots of differences vs. means of the body surface area (BSA) estimates obtained with the 3-dimentional optical imaging (3DOI) and with the following three predictive equations: Eq. # 5 by Shuter, Eq. # 9 by Kuehnapfel, and Eq. # 10 by Ashby-Thompson. In each plot, the solid horizontal line depicts the mean of the differences, whereas dashed horizontal lines represent the upper and lower limit of agreement (SD: standard deviation of the differences). The error bar displayed on each horizontal line represents the 95% confidence interval of the corresponding quantity. The dashed-dotted linear regression line (sandwiched between its 95% confidence interval curves) showed no proportional bias (its slope is not different from zero) for all equations. [file Image1.tif]
